# The future of ophthalmology and vision science with the Apple Vision Pro

**DOI:** 10.1038/s41433-023-02688-5

**Published:** 2023-08-04

**Authors:** Ethan Waisberg, Joshua Ong, Mouayad Masalkhi, Nasif Zaman, Prithul Sarker, Andrew G. Lee, Alireza Tavakkoli

**Affiliations:** 1 University College Dublin School of Medicine, Belfield, Dublin, Ireland; 2 Michigan Medicine, University of Michigan, Ann Arbor, MI USA; 3 Human-Machine Perception Laboratory, Department of Computer Science and Engineering, University of Nevada, Reno, Reno, NV USA; 4 Center for Space Medicine, Baylor College of Medicine, Houston, TX USA; 5 Department of Ophthalmology, Blanton Eye Institute, Houston Methodist Hospital, Houston, TX USA; 6 The Houston Methodist Research Institute, Houston Methodist Hospital, Houston, TX USA; 7 Departments of Ophthalmology, Neurology, and Neurosurgery, Weill Cornell Medicine, New York, NY USA; 8 Department of Ophthalmology, University of Texas Medical Branch, Galveston, TX USA; 9 University of Texas MD Anderson Cancer Center, Houston, TX USA; 10 Texas A&M College of Medicine, Bryan, TX USA; 11 Department of Ophthalmologys, The University of Iowa Hospitals and Clinics, Iowa City, IA USA

**Keywords:** Health care, Medical research

**Fig. 1:**
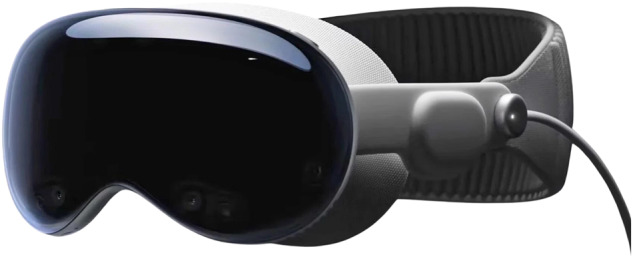
The Apple Vision Pro headset.

The Apple Vision Pro is an innovative gadget that integrates sophisticated technology to deliver an innovative spatial computing experience [1]. This device provides a seamless integration of digital media and the physical world and gives users the ability to navigate using their eyes, hands, and voice [1]. The device incorporates sophisticated cameras and sensors to facilitate precise visual perception, environmental awareness, and hand recognition [1]. The audio straps and speakers offer a superior ’Spatial Audio’ experience that integrates with surrounding ambient sounds. Most importantly, the Apple Vision Pro is fitted with personalized micro-OLED screens deliver a greater number of pixels to each eye, providing unique clarity (Fig. 1). Paired with eye-tracking technology and various other features, the Apple Vision Pro offers exciting technical advances with hopes of further improvements in performance and lower cost for extended reality devices in the near future.

The advent of extended reality has revolutionized the field of ophthalmology [2]. Current research includes utilizing extended reality for surgical training, ophthalmic diagnosis, and even monitoring of astronaut structural and functional vision changes during spaceflight [2–4]. With aging populations worldwide, vision screening will only become of greater importance in the coming years. Extended reality vision screening will also enable more frequent examinations of patients, which is particularly beneficial to those with chronic conditions like glaucoma and age-related macular degeneration (AMD) that have the potential to cause irreversible blindness if changes are left undetected [5]. Early detection, monitoring, and treatment of such diseases can potentially be an effective method prevent further vision loss [6]. The utilization of Apple Vision Pro and other future extended reality headsets could potentially be vital as a solution for vision screening globally, without needing any additional specialized equipment. In underserved areas and developing countries, there is a lack in trained ophthalmic healthcare experts and specialized equipment to screen populations, and extended reality visual screening can solve this deficit.

The potential integration of Apple Vision Pro’s sophisticated display technology also presents as a promising method to restore vision through augmented reality. For example, extended reality has previously been used to successfully been used to restore vision by reducing text metamorphopsia [7], visual field expansion [8], visual acuity [8], to being used to treat mild cases of strabismus [9]. All things considered, the Apple Vision Pro and future work in the extended reality space represent an innovative strategy for enhancing visual acuity and improving the overall quality of life of individuals suffering from ophthalmic disorders. Ophthalmologists and vision scientists may benefit from knowing of the unique technical capabilities of such technology and the advances in brings to the field.
